# Supplementary material for: Vulnerability and agency in research participants’ daily lives and the research encounter: A qualitative case study of participants taking part in scrub typhus research in northern Thailand
Source: PLoS One. 2023 Jan 25;18(1):e0280056. doi: 10.1371/journal.pone.0280056 (PMC9876277; doi:10.1371/journal.pone.0280056)
Supplement: S1 File — (DOCX) [file pone.0280056.s001.docx]

**Topic Guide for Interviews**

**Group 1**

Thank you so much for agreeing to participate in our REACH ethics study. I appreciate you taking time to speak with me. We are interested in finding out more about your thoughts, experiences, and views about participating in research studies and how this affects your daily life. The purpose of this project is to better understand how participating in different types of research can offer benefits, respond to your needs, or possibly add to challenges for women, children and families in Thailand. We will talk for about an hour, but you can stop at any time. You do not need to answer any questions which you don’t want to answer. It is fine to answer only what you are comfortable talking about. There is no right or wrong answer to these questions. We are interested in understanding more about your daily life and family and your experiences.

**Warm-up and daily life:**

To begin with I’m going to talk to a bit about yourself, your family, your daily life and your background.

1. Can you tell me a little about yourself and your family?
   1. How many siblings/ children do you have?
   2. How many people live in your household?
   3. Where are you from? Where were you born? [If they were born in a different place ask more about why did you move? What was happening at that time?]
2. Where do you live?
   1. How long have you lived here? How far is it from here? How do you come to this place?
3. Where did you go to school?
   1. *If little education:* can you tell me more about why you didn’t study?
   2. Has this had any effect on your life?
4. What languages can you speak, read or write?
   1. *If language barrier;* could you tell me more about that? What does this mean for your life? Job? Healthcare?
5. What are your current daily activities?
6. What do you do for a living? What are your family’s sources of livelihood? Do you have to send money home (if migrants)?

**Challenges, problems, difficulties in daily life**

Thank you. I’m going to ask you a few questions now about any challenges you or your family might have experienced now or in the past, as well as about any sources of support you have been able to make use of.

1. Would you describe any of the things we have discussed so far as challenges?
2. How are things going in your life in general?
   1. How would you describe your own and your family’s health?
   2. Do you eat and sleep well?
   3. Are there any concerns about your or your family members’ health?
3. When needed, where do you usually seek health care from?
   1. Why do you chose this/these place(s)?
   2. Do you seek health care from any other places? [Any choices, clinic, pharmacy, hospital, PCU, traditional healer]
4. Apart from medicine and information do the staff at this clinic/hospital/researchers do anything else to help you?
   1. If so, how have they helped you?
5. Have you faced any other challenges/difficulties/hardship in your life (economic, political, social and cultural)? Can you tell me more about that?
   1. If no real problems ask about neighbors’ problems
6. Are there any problems in your community that impact on you/ your family negatively or that you worry about?

**Sources of support, mitigating factors, coping, resilience:**

1. When you face some challenges/difficulties/hardship, what do you do? How do you cope with them?
2. Have you sought support from anyone such as your family members, friends, relatives, and people in your community etc.?
3. What are some of the main sources of support in your life for example for your health, for work, family matters, child’s education etc.?
4. What sources of support are most important to you and why?

**Research participation**

Thank you for telling a bit about your life and background. We’re now going to move on to talk about your illness and experience of the study

To help us understand what happened at each stage of your participation in the research, we have a diagram that I’d like you to look at *[show them the diagram]*. It shows some of the different stages we are interested in – starting from when you/ your child first became ill up until when you the study finished. We can look at the diagram together and I will ask you about what happened at each stage and how those experiences and activities made you feel.

When I ask you to tell me how you felt about something. If it’s helpful, you can point to one of the faces at the bottom of the page to show how you felt at that time. As you can see, these represent a range of emotions from happy to sad or angry. There is also one you can point to if you felt confused!

Can you describe your illness to me when you were admitted in the hospital with scrub typhus disease?

- What were your signs and symptoms?
- What do you think caused your illness?
- Where/ who did you go to for help/treatment before admission? Why?
- How long were you unwell before you went to the hospital?

Can you describe your journey to the hospital?

- Who took you to the hospital?
- How was the travelling?
  - Easy or difficult? Why? – Distance, Cost, Food, Weather etc.

What happened when you arrived in hospital?

- What were you told about your illness?
- *If not fluent in Thai* What language were they speaking? Did you have an interpreter? Did they have any difficulty explaining things to you? Were there times when no-one was there to translate for you? What happened?
- Did you understand what they were telling you?

How was the rest of your stay in the hospital?

- How did it make you feel?
- How many days did you stay in the hospital? Who took care of you when you were admitted in the hospital?
- Did the illness cause any other problems for you or your family? e.g. childcare, loss of work/ income

Treatment costs

- Did you have to pay for any of the treatment?
- What’s your health scheme/ insurance?
- Unexpected costs?

When did you first hear about the study?

- What were you told about it? (Nurse asked to take a blood test for scrub typhus)
- Was there someone there to interpret for you? Was it the same person?

Decision

- Do you remember being asked to take part in the study?
- Why did you decide to say yes? Did you consider any other benefits?
- Would you have been treated if you had said no?
- Would you have received the same treatment if you had said no?
- Who made the decision? [Alone, other people] Did you ask permission from someone else?
- Was it difficult to make the decision to participate? Why or why not?

Consent

- Do you remember signing a consent form?
- What did they explain to you about the form? (Did you know what you were signing for?)
- Was the form and explanation clear or difficult to understand? Was your interpreter with you?
- How did this process make you feel?

Did you have any questions about the research (START/ EXIST) during that time or maybe later?

- Did you find answers to your questions?

Can you describe to me what you had to do because you are part of the study?

- Blood test / Urine test: What for? How did you feel?
- Medication?
- Follow up visits?

How do you feel about the compensation? Do you know why you received it?

Overall how did taking part in research (START/ EXIST) make you feel?

How did participating in this research affect your life?

- Did it change your daily activities or your family’s? Eg work, childcare, time
- Did it create any problems for you or your family?
- What did you like? Benefits?
- What did you dislike?

**Wrap-up**

Thank you for sharing all those details with me. Before finishing, I have a couple more questions about your experiences overall.

1. Would you participate in another similar study? Why/ why not?
2. Do you have any ideas or suggestions for how the researchers can make participation easier for you and your family?

Thank you again, so much, for your time. This has been so helpful. Do you have any questions for us or anything else you would like to tell me?

-END-


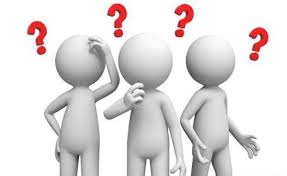
**What happened when….?**


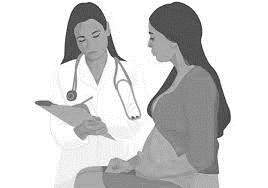


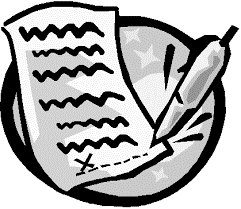

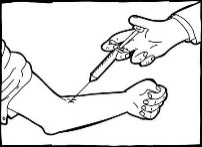

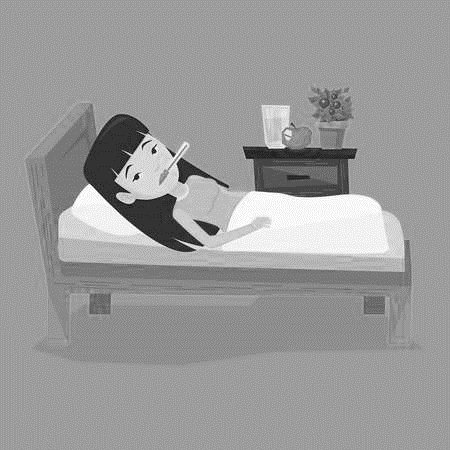

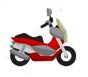


**How did that make you feel….?**


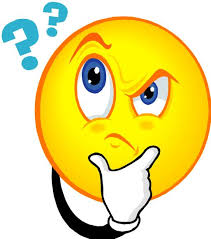

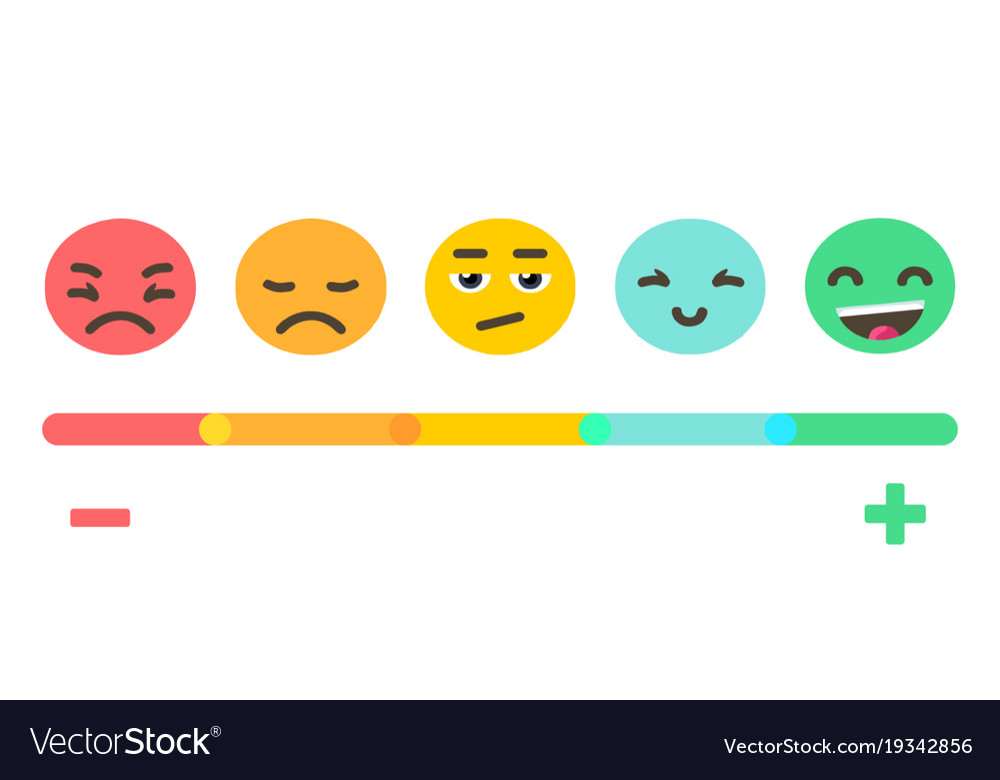


Research pathway diagram for participants
