# Supplementary material for: Vulnerability and agency in research participants’ daily lives and the research encounter: A qualitative case study of participants taking part in scrub typhus research in northern Thailand
Source: PLoS One. 2023 Jan 25;18(1):e0280056. doi: 10.1371/journal.pone.0280056 (PMC9876277; doi:10.1371/journal.pone.0280056)
Supplement: S2 File — (DOCX) [file pone.0280056.s002.docx]

*Resilience, Empowerment & Advocacy in Women’s and Children’s Health Research (REACH)*

**REACH Individual Interview Guide**

**Group 2 & 3**

Thank you for agreeing to participate in our REACH research ethics study interview. I know you are very busy so we really appreciate you taking time for this.

As mentioned, REACH is a study on research ethics in research involving vulnerable populations, with special focus on women, children and families participating in research. By research ethics we mean questions about what our ethical/ moral obligations are to participants, how we can improve understanding of research in consent, how we can best design studies to respond to participant needs and these sorts of questions. It can be challenging sometimes to know what to do when we are working with patients and participants who face many challenges in daily life, who struggle with money, jobs, or their health. We want to understand how research studies impact this experience for frontline healthcare workers and researchers. You can offer really valuable insights into how we can better improve participants’ experience of research and better support health workers whose patients are involved in research.

We will talk for about an hour. If we need to continue at another time we can schedule a follow-up appointment. You do not need to answer any questions which you don’t want to answer. It is fine to answer only what you are comfortable talking about. There is no right or wrong answer to these questions. Your perspective really matters to us, so we really want to hear what you think and what your experiences have been.

Do you have any questions about any of this, before we start?

**Section 1: Warm-up**

1. To start with, can you tell me a little about your professional background and the type of work you do? What does your day to day job involve?
2. Apart from working in this PCU/hospital, do you have other work/roles in the community?

**Section 2: Participants vulnerabilities, sources of support**

1. Can you tell me more about the patients you see – what health challenges do they face?
   1. Do you think people face any challenges accessing healthcare?
   2. Do you have any patients that not have documents/Thai ID? How does that affect their access to healthcare?
   3. Do you see people with mental health problems? What challenges can this cause them in their day to day lives? What services and support are available to them?
2. What kinds of challenges do your patients typically face in their daily lives?
   1. Prompt for social, economic
   2. Ask specifically about women and children
3. Are there any people or households that are more affected/at risk than others? In what way?
4. What would you say are the main sources of support for your participants? Where do they find help for daily health and social challenges? How do individuals and families cope with difficulties in daily life?
5. Do you ever personally feel responsible for helping patients or families with such difficulties, beyond treating them medically?

**Section 3: Benefits and burdens of research**

Thank you, we’re now going to move onto talking about research

1. Could you tell me about your experiences of research? What studies have you been involved in? What roles did you have?
2. What kinds of research studies are your patients involved in here in the clinic/ hospital?
3. How do research studies affect your day-to-day practice here? What are some of the benefits or burdens for the clinic/hospital?
4. We’re interested to know how participation in research affects patients and their families.
   1. Does participating in research create any positive impacts or benefits for the patients? What about for their families?
   2. Does participating in research cause any negative impacts or difficulties for patients? What about for their families?
   3. Do you think it affects the healthcare that they receive?
5. How do the communities in the area feel about the research studies happening here—what do you think they see as some of the benefits and burdens?
6. Do you think researchers/ research studies have any responsibilities for responding to kinds of health and social difficulties and daily challenges we discussed a minute ago?

**Section 4: Ethical situations that happen along the research pathway:**

Ok, thanks so much. Now I’d like us to shift our focus to any ethical challenges or situations that might come up in the context of research in this clinical setting. Times when you, other health workers or someone on the study team are not sure what the right thing to do is. An example would be issues around making sure research participants are fairly compensated for difficult journeys to the clinic. We will use this picture to help us think about ethical questions or situations that might come up at different stages of research. **Please remember that we are not conducting an audit of this clinic and would like you to feel free to share any challenges without worrying that it will reflect poorly on your colleagues or the clinic – challenges are a natural part of conducting research in any setting.** (Put up ppt slide, or give handout.)

1. Do any challenges arise at any of these points for the participants or for you as a healthcare worker?

Probes:

- 1. Can you say more about that? Who is most affected by this issue? In what way affected?

**Wrap-up**

1. Where along pathway do we see the most challenges? Where do we need more/different ethics support?
2. When you face ethical challenges or difficulties like the ones you have mentioned, do you as have any sources of support that you rely on to assist you?
3. What other support would be helpful to have in place for you and/or members of your team?
4. Overall, is there anything that could make the experience of participating in research better for you or your patients?

We have covered a lot of territory – is there anything else you would like to add, or any other thoughts about this general topic you would like to share with me? Thank you again, so much, for your time. This has been so helpful.

If we would like to follow up with you about specific insights you have shared today, for example, to clarify something, would it be okay for us to contact you again? Are you interested in hearing about the findings from our research ethics study?

Handout: Research Pathway

Share with researchers to prompt discussion around Q14

What ethical issues arise at each stage?

*Go through each step in the research pathway allowing the interviewee time to respond. There are some specific issues for each stage that you may want to ask about if they don’t naturally come up in the conversation. These are noted in the boxes on the right.*

**Research Pathway Diagram – Guide for interviewer with probes for answering Section 4, Q14**

**We know that you are usually not involved at this stage of the research pathway, but we have a couple of questions we’d like your views on as a frontline health worker:**

- Who usually decides what research takes place in your PCU/hospital?
- Can you explain a bit more about how that decision is made?
- Is there any input on what is studied and how studies designed by the participants? By the community?

**Could you tell us a bit more about your role in recruiting and enrolling patients?**

- What do you find challenging about this process?
- Are there any types of patient that are more likely to be enrolled in research than others? Any that are less likely?
- Do you see any challenges resulting from including some people into studies and excluding others?

**Now thinking about consent – could you tell me a bit about the process for consenting patients in your clinic?**

- Are there any parts of the process that create challenges for staff or patients?
- How much do you think participants understand about the consent process?
- One of the challenges we have found is that children often stay with their grandparents or aunties while their parents work in another place. How do you think we should take parental consent for those children? Are there times when it is appropriate to take consent from someone other than the parents?
- Why do you think people agree/ disagree to participate in these studies?
- How do individuals/ families make decisions to participate?
- What do you think about the compensation and incentives that are offered – are they fair? Do they affect people’s decisions in any way?

**Now when thinking about the study procedures:**

- Do any challenges arise for participants or staff in trying to fulfil the study procedures?
- Is it ever challenging to meet patients’ clinical needs whilst also meeting research objectives?
- Are there any differences in the standard of care, or the type of care received by patients compared to study participants?
- Do patients or staff ever notice a difference in the way research participants and patients are treated? (e.g. gifts, compensation) How does this make you feel?

**Post-study:**

- Do you see any challenges relating to the way studies close/ withdraw from participants?
- How often are findings shared with frontline staff?
