# Supplementary material for: Vulnerability and agency in research participants’ daily lives and the research encounter: A qualitative case study of participants taking part in scrub typhus research in northern Thailand
Source: PLoS One. 2023 Jan 25;18(1):e0280056. doi: 10.1371/journal.pone.0280056 (PMC9876277; doi:10.1371/journal.pone.0280056)
Supplement: Attachment — Submitted filename: Response to Reviewers.docx [file pone.0280056.s003.docx]

# Response to reviewers

*Many thanks for the time and attention you have given to our manuscript. We appreciate all the comments that have been made and have responded to each point below.*

Editor’s comments:

When submitting your revision, we need you to address these additional requirements.

1. Please ensure that your manuscript meets PLOS ONE's style requirements, including those for file naming. The PLOS ONE style templates can be found at 
[https://journals.plos.org/plosone/s/file?id=wjVg/PLOSOne_formatting_sample_main_body.pdf](https://protect-eu.mimecast.com/s/3CJ7CYQp4FzP1xHVHwGZ?domain=journals.plos.org) and 
[https://journals.plos.org/plosone/s/file?id=ba62/PLOSOne_formatting_sample_title_authors_affiliations.pdf](https://protect-eu.mimecast.com/s/W4G1CZwO5C3y4NSxAhuM?domain=journals.plos.org)

*The file names have been changed in line with the submission guidelines*

2. When reporting the results of qualitative research, we suggest consulting the COREQ guidelines: [http://intqhc.oxfordjournals.org/content/19/6/349](https://protect-eu.mimecast.com/s/CT5KC1Y15C7KzYf1wsDU?domain=intqhc.oxfordjournals.org). In this case, please consider including more information on the number of interviewers, their training and characteristics; and please provide the interview guide used.

*Additional details have been added to the Methods section.*

*The topic guides have been uploaded as supplementary material.*

3. We note that the grant information you provided in the ‘Funding Information’ and ‘Financial Disclosure’ sections do not match.

When you resubmit, please ensure that you provide the correct grant numbers for the awards you received for your study in the ‘Funding Information’ section.

*This has been verified and added to the cover letter.*

4. In your Data Availability statement, you have not specified where the minimal data set underlying the results described in your manuscript can be found. PLOS defines a study's minimal data set as the underlying data used to reach the conclusions drawn in the manuscript and any additional data required to replicate the reported study findings in their entirety. All PLOS journals require that the minimal data set be made fully available. For more information about our data policy, please see [http://journals.plos.org/plosone/s/data-availability](https://protect-eu.mimecast.com/s/n3zAC2g46hNAlwtXopuo?domain=journals.plos.org).

Upon re-submitting your revised manuscript, please upload your study’s minimal underlying data set as either Supporting Information files or to a stable, public repository and include the relevant URLs, DOIs, or accession numbers within your revised cover letter. For a list of acceptable repositories, please see [http://journals.plos.org/plosone/s/data-availability#loc-recommended-repositories](https://protect-eu.mimecast.com/s/VXb_C3jR7S5VAQiEMbBR?domain=journals.plos.org). Any potentially identifying patient information must be fully anonymized.

Important: If there are ethical or legal restrictions to sharing your data publicly, please explain these restrictions in detail. Please see our guidelines for more information on what we consider unacceptable restrictions to publicly sharing data: [http://journals.plos.org/plosone/s/data-availability#loc-unacceptable-data-access-restrictions](https://protect-eu.mimecast.com/s/9Vg3C4kmgIpG01U3cVYm?domain=journals.plos.org). Note that it is not acceptable for the authors to be the sole named individuals responsible for ensuring data access.

We will update your Data Availability statement to reflect the information you provide in your cover letter.

*See cover letter for more details*

5. Your ethics statement should only appear in the Methods section of your manuscript. If your ethics statement is written in any section besides the Methods, please move it to the Methods section and delete it from any other section. Please ensure that your ethics statement is included in your manuscript, as the ethics statement entered into the online submission form will not be published alongside your manuscript.

*The statement has been moved to the methods section*

Reviewers' comments:

Reviewer #1: This manuscript presents qualitative data about daily life challenges and how agency is exercised among a hill tribe ethnic minority in Thailand, particular as they relate to participation in biomedical research relevant to this group (i.e. scrub typhus studies). The manuscript is well-organized, clearly written and should be of some interest to researchers, research ethics committee members and ethicists interested in issues related to the inclusion of ‘vulnerable’ populations in research in low-and-middle income countries (LMICs). Major and minor comments are detailed below.

Major comments

A possible criticism of the manuscript is that it does not seem to provide much new information to the ongoing discussion about biomedical research in LMICs with marginalized communities. It has long been known that social determinants adversely impact health as well as willingness/ability to participate in research that might benefit such communities. On the other hand, it is likely that such a descriptive account has not been done before with this particular community, and certain barriers (particularly linguistic, citizenship status and access to health care benefits) may resonate with experiences of other communities in LMICs.

The methods section (lines 126-134) does not really convey the methods. Only by line 180 is it clear that semi-structured individual interviews were conducted. There are many ways of doing case studies, so what did ‘case study design’ involve here? There is little sense of what kinds of questions were asked to the three different groups, including whether they were asked the same sorts of questions in order to make comparisons.

*The Methods section has been renamed ‘Study setting and methods’ to better reflect the included material. This allows for a separate Methods section which is now more focused.*

*Further explanation has been added about the case study design (lines 168-171) and topic guides (203-210). The topic guides will be submitted as supplementary materials.*

It would seem that although two different research studies were involved, the results (in the form of illustrative quotes or descriptions) are lumped together. This makes it hard to know when (for example) people talk about future benefits of research or personal benefits, they are talking about the observational study or the randomized controlled trial.
The participants make up the bulk of the respondents. In the reporting of the results, they also seem to be lumped together, i.e. it is unclear which of the participants participated in the observational study or the randomized controlled trial, or both. This way of reporting may conceal differences in experiences of participating in these studies.

*The reported benefits and burdens of taking part in research were remarkably similar between the 2 studies so have been grouped together. But it was an oversight not to add this fact to the results. It has now been added.*

*For clarity, the scrub typhus study (observational or RCT) has been added to the description of the quotes*

Although three groups were interviewed, the perspectives of research participants and family members are disproportionately respresented. Is this intentional? I counted 16 illustrative quotes from research participants and their family members, while there was only 1 from group 2, and 1 from group 3. The views of those in groups 2 and 3 seem to be reported in aggregate, with little indication of tensions or differences of opinion between group members or with the views of participants (perhaps there was a lot of consensus?).

*This manuscript does intentionally focus on the research participants views and this has now been added to the results section-line 255-257. Groups 2 & 3 have been used to triangulate the results. An early project title was ‘in their own voices’ and we have tried to illustrate the results with quotes from participants where possible. We have however switched one of the quotes from a group 1 to group 3 member (lines 336-339). There was a lot of consensus in the responses from groups 2 & 3 so they have not been separated.*

Was the formal network of translators established as a result of the qualitative study reported in this manuscript or was it already established prior to that? It is hard to tell from lines 568-571.

*Yes it was formed as a result of this qualitative study while the scrub typhus studies were still ongoing. The text has been clarified:*

*‘In response to this challenge a formal network of translators was established and trained during our data collection phase. At the time of writing the linked clinical studies are ongoing allowing the translators to be on stand by for participants unable to speak Thai fluently, rather than relying on informal translators (e.g. friends or family members) as is standard practice locally.’*

In the conclusion section, where it says research needs be designed to limit the burdens and respond to unexpected vulnerabilities (lines 641-643), it may be advisable to say something about approaches to do that, particularly in the context they describe. Would it include social science research in advance of the implementation of biomedical trials? Some form of community engagement? Something else?

*Additional text has been added to the discussion (lines 656-668) and conclusions (lines 691-692) suggesting ways in which this can be achieved.*

Minor comments

What is meant by ‘linked’ when the two studies are described as linked?

*It is referring to the scrub typhus studies which we have connected to in order to form the case study and draw research participants from.*

Line 542: “Overall, people …” Does this mean all interviewees across the three groups interviewed? This comes in after describing research participant views, so please clarify.

*This was referring to all participants and has now been specified.*

Line 80: should probably be “while some may be protected, others could be unfairly …”

*Comma added- thanks*

Lines 352-354: there seems to be either extra words or words missing in this sentence. Please recheck.

*Further explanation has been added:*

*‘There was one example of someone who was entitled to UHC but did not receive it because they could not afford the cost of travel to the administration office to register their house move, so had to pay the hospital fees (approximately 50 GBP, UHC entitlements are linked to your place of residency).’*
