# Supplementary material for: Vulnerability and agency in research participants’ daily lives and the research encounter: A qualitative case study of participants taking part in scrub typhus research in northern Thailand
Source: PLoS One. 2023 Jan 25;18(1):e0280056. doi: 10.1371/journal.pone.0280056 (PMC9876277; doi:10.1371/journal.pone.0280056)
Supplement: Attachment — Submitted filename: Response to Reviewers.docx [file pone.0280056.s004.docx]

# Response to Reviewers

*Many thanks to the reviewers for taking the time to review our manuscript and for giving your helpful feedback and comments. We’ve responded to each comment below.*

Reviewer #2: Manuscript #: PONE-D-21-20925R1
Topic: Vulnerability and agency in research participants’ daily lives and the research encounter: A qualitative case study of participants taking part in scrub typhus research in northern Thailand.
Academic Editor: Dr. Ahmed Mancy Mosa
Thanks very much, Dr. Ahmed Mancy Mosa, for giving me the opportunity to review and give comments. My thanks also go to the authors of the manuscript for their interest to deal with this interesting topic addressing the issue of vulnerability and agency in research participants which are the areas that need more research to come up with concrete and convincing shreds of evidence. I am impressed with the title, and the way the authors synthesize and present the result. The whole section of the manuscript has been well prepared and comes up with interesting findings. In fact, this is the second version of the corrected manuscript based on the first comments given by the other reviewers, the authors come up with a well-organized and appropriately synthesized document. I have only one question as well as a suggestion to be addressed by the authors before the manuscript will be published. What are the clinical and public health implications of this research? Please, incorporate the clinical, and public health implications of the findings of this research.

*These have been added to the discussion section.*

Reviewer #3: Thanks to the authors for showing us the daily vulnerabilities and initiatives of participants in scrub typhus research in northern Thailand and how they interact with participants' own research experiences. Overall, this manuscript is interesting. But there are plenty of concerns.

1. The implications of the study are unclear. The extensibility and extensibility of the research are not enough.

*A discussion of the transferability of the study has been added to the strengths and limitations section. The implications have been made clearer in the discussion section.*

2. The definition of some words in the manuscript is not clear, which will cause unnecessary trouble to readers. For example, what does “agency” mean in the title and START study and EXIST study in Table 1?

*Agency is explained in the introduction including a definition which is now placed in parentheses for additional clarity (line 90-92 clean version)*

*We have removed the acronyms of the study titles and revised the table accordingly.*

3. Key words: The keyword global health research is not reflected in the manuscript.

*We have removed this keyword.*

4. Introduction: There's so much in this section, it's more like a review. It is recommended to simplify.

*We have simplified this section while still giving an overview of the concepts of vulnerability and agency.*

Study setting and methods:
a) A large number of references to scrub typhus and related studies are not recommended for inclusion in this manuscript.

*Several references have been removed.*

b) Tables 2 and 3 are recommended for the results section. The obvious Table 3 describes the demographic characteristics of the first group of subjects. Why does it not reveal the most basic demographic trait, sex? Why not describe the demographic characteristics of the subjects in the second and third groups? The grouping of Education, Employment and Legal status in Table 3 is not clear. For example, Primary school or less and None in Education may coincide, studying refers to students in Employment? What does "Right to remain" mean in Legal status?

*Tables 2 & 3 have been moved to the results section as suggested.*

*Sex has been added to table 3. It was not there originally as we only interviewed female and children research participants. Not all of the variables are relevant for group 2 & 3 but their basic demographics have been added to table 3.*

*Education & employment: some categories have been renamed for clarity.*

*Legal status: right to remain means they are not Thai citizens/nationals but can stay in Thailand as long as they choose. Similar to indefinite leave to remain in The UK. More details are given in the introduction 126-128 and a footnote has been added to table 3.*

5. Results:
a) This study adopts thematic analysis, but it is difficult to see the inductive theme in the results.

*While we identified various potential inductive themes such as ‘hopes and aspirations for the future’ these were merged under the final theme ‘coping and agency in daily living’ during the analysis process.*

b) Lines 253-258 seem to describe the purpose of the study and are suggested to be put in the background.

*The sentence starting ‘we intentionally focused…’ has been moved to the methods section. The sentences started ‘ to understand the wider context…’ has been kept as way of introduction to the results section.*

c) The responses of the other two groups were used to support the analysis of the first group. For example, in line 325-339, the first group is analyzed but the answers of the third group are used as evidence.

*Apologies that quote was swapped as part of the first review. We have clarified that these findings were corroborated by Group 3.*

d) Lines 385 to 387, using the two nurses as an example, are not supported by evidence.

*An illustrative quote has been added*

e) The contents of Benefits of research and Burden of research do not seem to have much relevance to this study, but more to describe the benefits and burdens of the previous two studies on scrub typhus.

*One of the study aims was to understand how participants’ vulnerabilities and agency affected their experience of taking part in research. By drawing on their experiences of scrub typhus research participation we were able to explore their lived experiences rather than asking them hypothetically about benefits or burdens they may have from taking part in research. It would be too soon to ask them about the benefits and burdens of taking part in this qualitative study- which for most respondents was a single interview.*

6. Discussion:
a) The authors suggest redefining vulnerability. However, there are no more comments and suggestions in the discussion section.

*While we do not give a new definition for vulnerability we have made some suggestions about how thinking should change and practical steps that researchers and ECs can take to do this. This can now be found under the implications heading. We have deleted text that imply we are redefining vulnerability.*

b) The examples on lines 588-591 do not fit in the discussion section.

*The example has been deleted*

7. References: References that are too old are of little value, especially 6-9 and 11

*Thank you for this observation. References 6 & 9 have been removed. The remaining references mentioned date from 2004-2009. While they are on the older end of the spectrum we feel they are key references on vulnerability, have not been replaced by newer manuscripts, and still provide valuable insights into vulnerability and the thinking of the research community.*
